# Distinguishing Human Peripheral Blood NK Cells from CD56^dim^CD16^dim^CD69^+^CD103^+^ Resident Nasal Mucosal Lavage Fluid Cells

**DOI:** 10.1038/s41598-018-21443-5

**Published:** 2018-02-21

**Authors:** Meghan E. Rebuli, Erica A. Pawlak, Dana Walsh, Elizabeth M. Martin, Ilona Jaspers

**Affiliations:** 1 Curriculum in Toxicology, University of North Carolina at Chapel Hill, Chapel Hill, NC USA; 2 Center for Environmental Medicine, Asthma, and Lung Biology, University of North Carolina at Chapel Hill, Chapel Hill, NC USA; 3 Department of Environmental Sciences and Engineering, Gillings School of Global Public Health, University of North Carolina at Chapel Hill, Chapel Hill, NC USA; 4 Department of Pediatrics, University of North Carolina at Chapel Hill, Chapel Hill, NC USA

## Abstract

Natural killer (NK) cells are members of the innate lymphoid cells group 1 (ILC1s), which play a critical role in innate host defense against viruses and malignancies. While many studies have examined the role of circulating peripheral blood (PB) CD56^+^ NK cells, little is known about the resident CD56^+^ cell population. Therefore, matched CD56^+^ cells from nasal lavage fluid (NLF) and PB of smokers and non-smokers were compared phenotypically, via flow cytometry, and functionally, via NK-cell specific gene expression. NLF and PB CD56^+^ cells had similar expression of CD56, but differentially expressed tissue residency (CD69 and CD103) and cytotoxicity (CD16) markers. In addition, NLF CD56^dim^ cells expressed lower levels of cytotoxicity-associated genes, perforin (*PRF1*) and granzyme B (*GZMB*), and increased levels of cytokines and cell signaling molecules, *TRAIL*, *IFNGR2*, and *IL8*, as compared to PB CD56^dim^ cells. In smokers, *ITGA2* was downregulated in NLF CD56^dim^ cells, while markers of cytotoxic function were primarily downregulated in PB CD56^dim^ NK cells. Overall, NLF CD56^dim^ cells are a unique cell population that likely play a role in orchestrating innate immune responses in the nasal cavity, which is distinct from their role as a non-antigen-restricted cytotoxic CD56^dim^ lymphocytes in the PB.

## Introduction

Natural killer (NK) cells belong to the family of innate lymphoid cells (ILCs), sharing many characteristics with ILC group 1 (ILC1) in non-mucosal tissues^1^, including the expression of certain surface markers and the ability to produce interferon gamma (IFNγ) in response to invading pathogens. In mucosal tissues NK cells are thought to be grouped with ILC1 cells such as Intraepithelial ILC1s (ieILC1s) due to their similarity in activation and function^2^. ieILC1s are particularly similar to NK cells in that they also express CD56^3^ and resemble NK cells’ function as one of the body’s first lines of defense against viral infections and cancer development through their cytolytic capacities, releasing perforin and granzyme B, as well as through coordination of innate immune response through cytokine production^4–6^. In addition, NK cells and ILC1 share roles in immunosurveillance and orchestration of the adaptive immune response through promotion of antigen presentation as well as cytokine production^1,7,8^. In humans, NK cells and ieILC1s are generally defined phenotypically by the expression of CD56 and lack of CD3 surface markers with ieILC1s also expressing additional markers of tissue residency, such as CD103 or CD69^1^. CD69 and CD103 are involved in retaining lymphocytes within local tissues, providing markers for tissue resident lymphocytes as they are not present on circulating NK cells^9^. The origin of tissue resident NK cells, whether they are derived from either lymph nodes or the blood, or whether, similar to lung macrophages, they represent a resident cell type^10,11^, is not well understood^12^.

The majority of what is known about NK cells is derived from studies on peripheral blood (PB) and lymphoid NK cells as they are highly abundant and easy to access. In conventional NK cells, additional surface markers, such as CD16 indicate different functional subtypes, such as cytotoxic NK cells being CD56^dim^CD16^+^ and cytokine-producing NK cells being predominantly CD56^bright^CD16^−^, though recent studies have identified more complex and nuanced roles for NK cell subtypes in the PB^13^. Unique tissue-specific NK cell populations have been described in the skin, uterus, intestine, and liver^14,15^, indicating that NK cells are far more diverse than what is represented in circulating NK cells, but how much these populations overlap with ieILC1s is not clear. In the respiratory tract, ILC1s and NK cells are present in the mucosa^12,16,17^. The airway mucosa provides the first line of defense against pathogens and environmental toxins and therefore is a logical location for both ILC1s and NK cells to coordinate innate and adaptive immune responses to any foreign onslaught. Several recent studies have defined the phenotype of human lung resident NK cells^9,18^, while less is known about tissue resident nasal NK cells or ieILC1s in the nasal mucosa. NK cells comprise about 10% of the lung lymphocyte population^19^ and are primarily CD56^dim^ with low abundance of the CD69 tissue resident marker, which is thought to be due to the vascularity of the lung and high circulation of NK cells from the blood into the lung^9^. In contrast, we have previously demonstrated that in the nasal mucosa, which is predominantly macrophage-free, about 23% of non-squamous cells are CD56^+^ cells, with both CD16^bright^ and CD16^dim^ populations of CD56^+^ cells being present following viral infections^16^. Cytokine-secreting CD56^dim^CD16^dim^ cells appear to be more prevalent than cytotoxic CD56^dim^CD16^bright^ cells at baseline^16^, but little is known in regards to markers of tissue residency. We have also demonstrated that superficial scrape biopsies of the nasal mucosa contain CD45^+^CD56^+^ cells, but based on histological evaluations, it is unclear whether these cells are embedded in the tissue or are collected as part of leukocytes populations patrolling the nasal mucosa^20^. In addition, it is unknown how nasal resident CD56^+^ cells differ from peripheral blood NK cells and to what extend these cells express CD16 and/or CD69 and CD103, thus potentially differentiating them as NK cells or ieILC1s.

Cigarette smoke is known to suppress innate immune functions^21^ and we have previously shown that CD56^+^ cells in the nasal lavage of smokers show reduced markers of cytotoxic activation following viral infection^16,22^. Cigarette smoke-induced immune suppression has specifically been shown in NK cells *in-vitro*, yet far less is known in humans *in-vivo*. To further understand how nasal CD56^+^ cells differ from PB CD56^+^ NK cells and whether smoking induces functional changes in these cell populations we designed the following study: PB and nasal lavage CD56^+^ cells were isolated from healthy subjects, enriched, and either phenotyped using flow cytometry or gene expression profiling. Smoking-induced changes were further assessed comparing PB or nasal lavage CD56^+^ cells via gene expression profiling. Our results indicate that resident nasal CD56^+^ cells are phenotypically distinct from PB CD56^+^ NK cells and express markers more consistent with ieILC1s. We also show that smoking reduces markers of cytotoxic activation in PB CD56^+^ NK cells, suggesting systemic immunosuppression, but smoking has little effects on nasal CD56^+^ cell gene expression profiles. Overall results suggest that nasal resident CD56^+^ cells are a unique cell population, resemble previously described ieILC1s, are phenotypically and functionally distinct from PB and lung resident NK cells, and express markers that are indicative of a primarily immune orchestrating role in the nasal mucosa.

## Results

### NK Cell Enrichment

**Figure 1:**
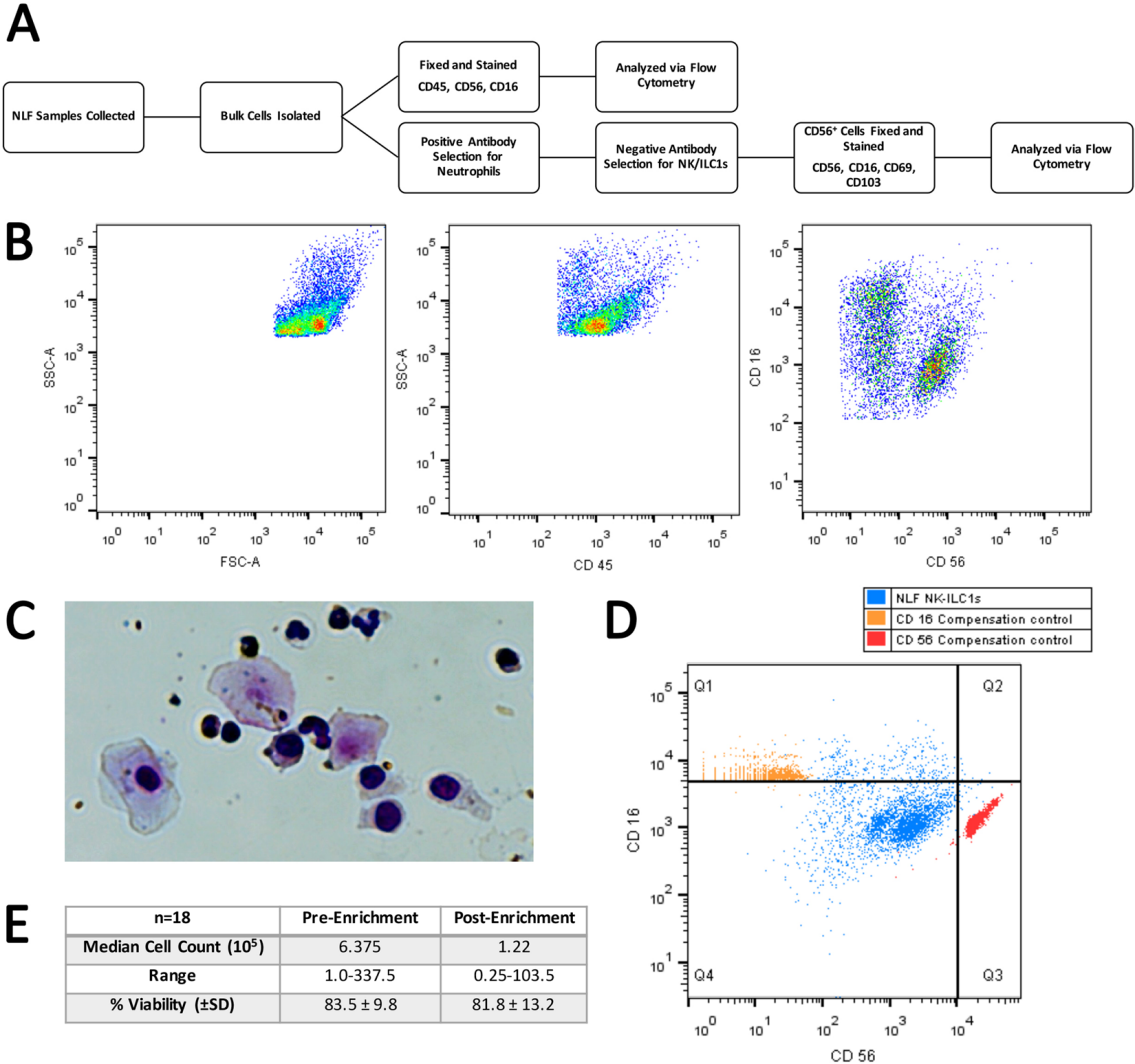
Isolation and phenotyping of NLF CD56^+^ cells. (**A**) Flow chart of experimental methods to validate CD56^+^ NK/ILC1 enrichment (**B**) Bulk NLF cell scatter properties, and stained for CD45, CD16, and CD56, (**C**) Diff-quick stain of adherent cells post- CD56^+^ cell enrichment procedure, demonstrating that the remaining adherent cells are a mixture of epithelial cells, neutrophils, and other non-NK cells, leaving an enriched CD56^+^/NK cell population in the supernatant. (**D**) CD56/CD16 phenotyping of post-enrichment NLF cell samples: Enriched NLF NK/ILCs (blue), CD16 compensation control (yellow), CD56 compensation control (red). All plots/images are representative of n = 3 individuals. (**E**) Median cell counts and viability pre- and post-enrichment, n = 18 individuals. Mean ± SD.

As described previously^16^, based on the expression of CD45, CD16, and CD56, NLF leukocytes are comprised of two major distinct populations, neutrophils (CD56^−^CD16^+^) and CD56^+^ NK cells (CD56^+^CD16^+/−^) (Fig. 1B, Supplementary Table 1). In order to further characterize these nasal CD56^+^ cells based on their gene expression profiles, it was necessary to enrich the NLF population for the specific cell population of interest, CD56^+^ NK cells. Figure 1C shows that the cells selected by the negative NK cell selection antibody cocktail were predominantly squameous epithelial cells, epithelial cells, and neutrophils. The resulting supernatants containing the CD56^+^ cell enriched mixture were determined to be 90% CD56^+^, but dim for both CD16 and CD56 (Fig. 1D). Further analysis of cell number and viability indicated that our NLF NK cell enrichment protocol sufficiently enriched nasal NK cells for subsequent cell type-specific gene expression analysis to assess NK cell function (Fig. 1E).

### Comparison of PB and Nasal Resident NK Cell Surface Markers

**Figure 2:**
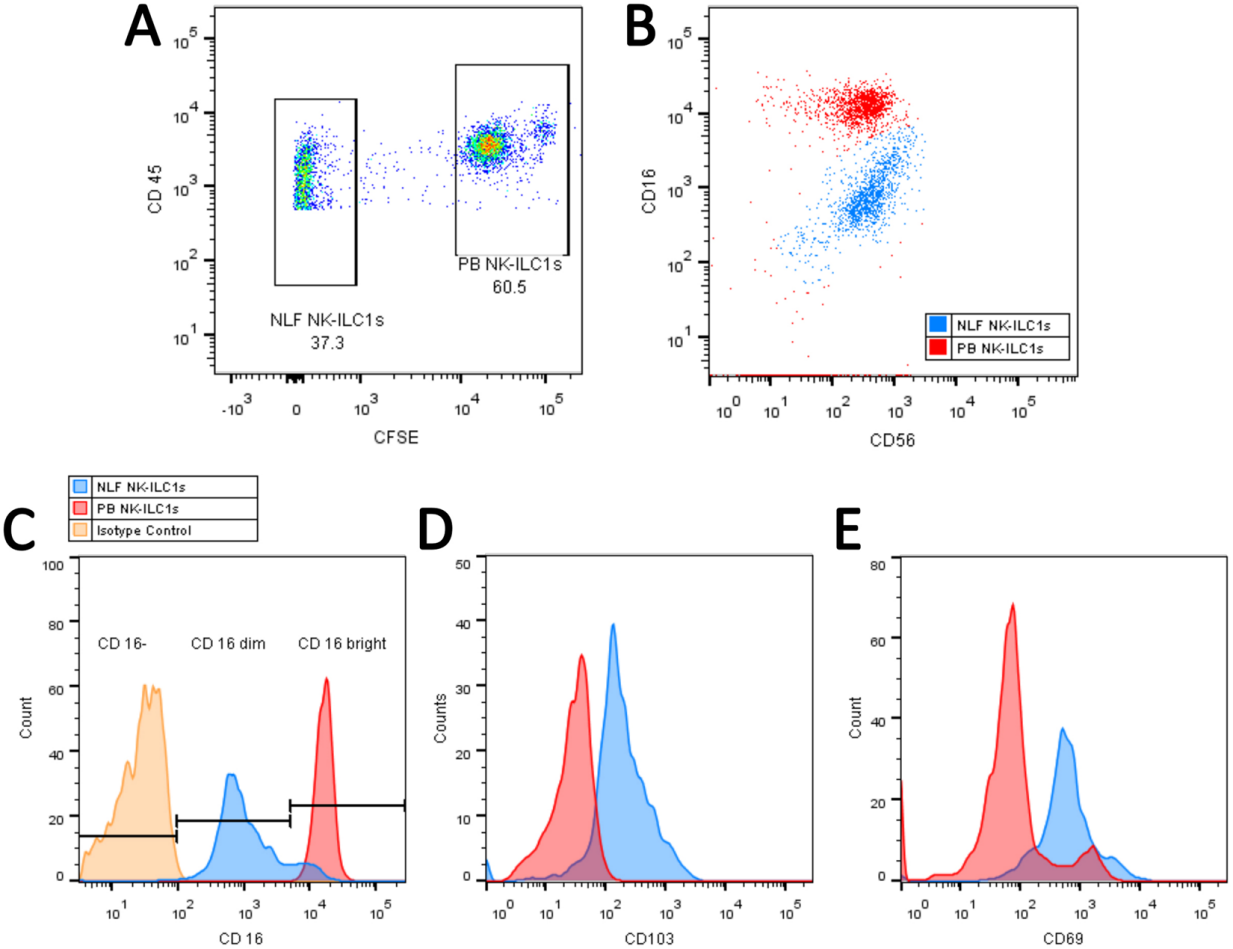
Comparison of surface marker expression on NLF versus PB CD56^+^ cells. (**A**) CD45^+^ CD56^+^ cells stratified into NLF CD56^+^ cells (CFSE^−^ (unlabeled)) and PB CD56^+^ cells (CFSE^+^, labelled), (**B**) CD16 and CD56 expression in CFSE^+^ (PB) and CFSE^−^ (NLF) CD56^+^ cells, (**C**) Surface expression of CD16, (**D**) CD103, and (**E**) CD69 in isotype controls (yellow), NLF CD56^+^ cells (blue) and PB CD56^+^ cells (red).

To determine differential surface marker expression in NLF and PB CD56^+^ NK/ILC1 cells from the same subjects, we labeled PB CD56^+^ NK/ILC1 cells with CFSE. CFSE-negative resident NLF CD56^+^ NK/ILC1 cells could be clearly distinguished from CFSE^+^ PB CD56^+^ NK/ILC1 cells (Fig. 2A). Using CFSE labeling to distinguish NLF and PB CD56^+^ NK/ILC1 cells from the same subject, equal numbers of cells from each compartment were stained, fixed, and acquired together to assess relative expression of surface CD16 and CD56 markers in NLF and PB CD56^+^ NK/ILC1 cells (Fig. 2B). Surface marker expression indicates that both NLF and PB CD56^+^ NK/ILC1 cells were CD56^dim^, with all PB CD56^+^ NK/ILC1 cells also expressing high levels of CD16 (CD16^bright^). NLF CD56^+^ NK/ILC1 cells can be subdivided into a small population of CD16^bright^ cells (8.9% ± 1.2), expressing CD16 at similar levels seen in the PB CD56^+^ NK/ILC1 cells, and a majority of CD16^dim^ cells (90.7% ± 1.1) cells (Fig. 2B). Based on the surface marker expression and inclusion of compensation and isotype controls (Fig. 2C), we determined that NLF CD56^+^ NK/ILC1 cells are CD16^dim/+^, but show less relative expression of CD16 than the CD16^bright^ expression seen in PB CD56^+^ NK/ILC1 cells (p ≤ 0.03) (Fig. 2C). Based on these findings, we labeled NLF CD56^+^ NK/ILC1 cells as CD16^dim^. NLF CD56^+^ NK/ILC1 cells also expressed both CD103 (Fig. 2D) and CD69 (Fig. 2E) in comparison to no/low expression of this tissue residency marker in PB NLF CD56^+^ NK/ILC1 cells.

### Nanostring Based Gene Expression

#### PB vs NLF CD56+ NK/ILC1 cells

To determine more broadly phenotypic differences in NLF and PB CD56^+^ NK/ILC1 cells, we developed a gene expression panel, consisting of 64 genes related to NK cell function, including surface markers/receptors (n = 28 genes), mediators (n = 16 genes), signaling (n = 14 genes), markers for other potential cell populations (i.e. neutrophils, innate lymphoid cells, and macrophages, n = 6 genes), and housekeeping genes (n = 4 genes) (Supplementary Table 2). 7 genes were more robustly expressed in the NLF CD56^+^ NK/ILC1 cells than PB CD56^+^ NK/ILC1 cells (Table 1). The five genes showing the greatest differential expression in NLF and PB are Integrin Subunit Alpha 2/CD49b (*ITGA2*; fold change: 47.59, p < 0.001), interleukin-8 (*IL8*; fold change: 29.58, p < 0.001), CD14 (*CD14*; fold change: 15.67; p < 0.001), interferon gamma receptor 2 (*IFNGR2*; fold change: 11.66, p < 0.001) and Fc gamma receptor II-a/CD32 (*FCGR2A*; fold change: 4.56, p < 0.05). In contrast, 46 genes were more highly expressed in PB CD56^+^ NK/ILC1 cells as compared to NLF CD56^+^NK/ILC1 cells (Table 2), with the top five genes being perforin (*PRF1*, fold change; −1209.98, p < 0.001), granulysin (*GNLY*; fold change: 692.72, p < 0.001), CD247 (*CD247*; fold change: 469.09, p < 0.001), killer cell lectin like receptor/NKG2D (*KLRK1*; fold change: 357.94, p < 0.001), and killer cell lectin like receptor B1/CD161 (human equivalent of the mouse NK 1.1., *KLRB1*; fold change: 357.94). In addition, several markers of classical NK cell cytotoxicity were highly expressed in PB CD56^+^ NK/ILC1 cells, including granzyme B (*GZMB*, fold change: 71.37, p < 0.001) and natural cytotoxicity receptor 1/NKp46 (*NCR1*; fold change 65.27, p < 0.001) (Table 2). These gene expression data confirm our surface marker findings, where NLF CD56^+^ NK/ILC1 cells lacked the expression of conventional NK cells present in PB.

**Table 1:** Upregulated Gene Expression of CD56^+^ NLF cells.

Gene	NLF Count	PB Count	Fold Change	P-Value
*MAPK3*	737.58 ± 233.66	465.5 ± 84.12	1.58	0.001
*TNFSF10 (TRAIL)*	1521.82 ± 514.29	510.35 ± 162.18	2.98	<0.001
*FCGR2A (CD32)*	613.52 ± 1415.34	134.5 ± 303.19	4.56	0.02
*IFNGR2*	854.18 ± 285.21	73.29 ± 147.16	11.66	<0.001
*CD14*	1706.79 ± 2893.18	108.93 ± 344.98	15.67	<0.001
*IL8*	64629.39 ± 109689.28	2184.85 ± 17534.08	29.58	<0.001
*ITGA2*	296.28 ± 202.97	6.23 ± 8.59	47.59	<0.001

**Table 2:** Upregulated Gene Expression of CD56^+^ PB cells.

Gene	NLF Count	PB Count	Fold Change	P-Value
*JAK2*	436.7 ± 233.26	753.53 ± 136.45	1.73	<0.01
*STAT5A*	81.96 ± 44.9	144.75 ± 31.75	1.77	<0.05
*PTPN11*	295.93 ± 147.57	592.1 ± 88.86	2	<0.01
*STAT5B*	495.18 ± 557.79	1068.12 ± 180.67	2.16	<0.05
*SDHA*	334.67 ± 279.49	795.21 ± 127.36	2.38	<0.001
*TBP*	44.65 ± 19.23	107.1 ± 33.97	2.4	<0.001
*PTPN6*	332.24 ± 381.85	806.27 ± 194.78	2.43	<0.05
*TYROBP*	3628.97 ± 5616.2	9846.55 ± 2041.06	2.71	<0.05
*IFNAR2*	659.83 ± 279.36	1959.47 ± 365.08	2.97	<0.001
*MAPK1*	586.49 ± 259.42	1820.18 ± 288.58	3.1	<0.001
*MIP-1b*	998.18 ± 5811.49	3350.43 ± 303.32	3.36	<0.05
*CD69*	370.29 ± 2673.26	1555.12 ± 615.33	4.2	<0.05
*CXCR3*	19.45 ± 27.32	109.6 ± 37.08	5.64	<0.01
*FCER1G*	928.64 ± 1269.46	5664.64 ± 1055.06	6.1	<0.001
*IFNG*	16.46 ± 63.68	106.93 ± 79.18	6.5	<0.01
*IL18R1*	73.05 ± 47.25	561.34 ± 122.68	7.68	<0.001
*JAK1*	794.08 ± 456.51	6320.23 ± 822.64	7.96	<0.001
*KIR2DL4*	11.43 ± 34.12	103.07 ± 148.13	9.02	<0.01
*ITGB2*	808.51 ± 1451.66	7919.5 ± 1943.25	9.8	<0.001
*ITGAM*	149.92 ± 240.55	1991.81 ± 259.41	13.29	<0.001
*CD57*	10.08 ± 17.13	148.93 ± 72.98	14.77	<0.001
*IL12RB1*	12.86 ± 17.87	213.55 ± 44.86	16.6	<0.001
*FCGR3A*	440.68 ± 4818.37	8549.23 ± 3288.24	19.4	<0.001
*KIR2DS2*	24.99 ± 37.13	586.43 ± 1017.48	23.47	<0.001
*ITGAL*	132.94 ± 192.54	3449.6 ± 1329.2	25.95	<0.001
*KIR2DL2*	4.53 ± 7.43	123.72 ± 128.23	27.31	<0.001
*KLRD1*	24.84 ± 35.73	699.68 ± 512.4	28.17	<0.001
*FASLG*	15.44 ± 36.17	437.37 ± 144.66	28.32	<0.001
*KIR3DL2*	11.72 ± 17.3	429.23 ± 350.64	36.62	<0.001
*HCST*	173.41 ± 168.05	6922.24 ± 1082.04	39.92	<0.001
*SH2D1B*	147.13 ± 172.92	6242.69 ± 1152.3	42.43	<0.001
*NCR3*	8.17 ± 22.57	454.05 ± 83.84	55.55	<0.001
*KLRC1*	18.65 ± 43.55	1117.33 ± 590.64	59.9	<0.001
*NCR1*	7.93 ± 16.13	517.48 ± 80.94	65.27	<0.001
*GZMB*	201.51 ± 167.85	14380.42 ± 2535.39	71.37	<0.001
*KLRC2*	28.05 ± 73.67	2511.14 ± 1382.37	89.52	<0.001
*NFATC2*	35.08 ± 46.08	3145.71 ± 422.0	89.68	<0.001
*NCAM1*	17.08 ± 21.87	1607.37 ± 306.53	94.11	<0.001
*CD244*	23.1 ± 37.54	2334.19 ± 398.69	101.06	<0.001
*RANTES*	59.73 ± 59.73	9268.96 ± 2870.14	155.17	<0.001
*KLRC3*	8.33 ± 9.51	1597.82 ± 1842.55	191.84	<0.001
*KLRB1*	40.33 ± 39.52	8385.18 ± 1995.79	207.94	<0.001
*KLRK1*	28.04 ± 37.95	10038.25 ± 1941.62	357.94	<0.001
*CD247*	22.48 ± 35.79	10545.92 ± 1355.46	469.09	<0.001
*GNLY*	46.56 ± 33.92	32255.74 ± 12090.86	692.72	<0.001
*PRF1*	31.88 ± 40.84	38575.12 ± 4162.84	1209.98	<0.001

Pathway analysis was conducted using Gene Set Enrichment Analyses (GSEA) on differentially expressed genes in PB and NLF. PB CD56^+^ NK/ILC1 cells were eniched for genes that overlap with canonical natural killer cell mediated cytotoxicity pathways (28 genes, FDR q-value = 6.22E-57) and immune system pathways (30–32 genes, FDR q-value = 5.74E-38 to 8.24E-31). In addition there were significant overlaps with previous studies documented in the GEO database^23^ studying NK or CD8^+^ cytotoxic cells (Supplementary Table 3). However, nasal CD56^+^ NK/ILC1 cells were enriched for fewer genes in canonical natural killer cell mediated cytotoxicity pathways (3 genes, FDR q-value = 1.07E-03) and more closely aligned with other pathways and gene sets associated with downregulation in CD8^+^ cytotoxic cells and upregulation in monocytes. (Supplementary Table 4). These data further emphasize that NLF CD56^+^ NK/ILC1 cells differ significantly from traditional NK cells present in the PB, and do not align well with a cytotoxic phenotype.

#### Cigarette Smokers vs Nonsmokers

To determine whether and how smoking modifies NLF and PB CD56^+^ NK/ILC1 cells, we enriched NLF and PB CD56^+^ NK/ILC1 cells from current smokers (n = 9) and assessed gene expression profiles. Surprisingly, only one gene was differentially expressed in NLF CD56^+^ NK/ILC1 cells between smokers and non-smokers. *ITGA2* was significantly enhanced in NLF CD56^+^ NK/ILC1 cells from smokers (fold change: 2.05, p ≤ 0.05) (Table 3). Similarly, in PB CD56^+^ NK/ILC1 cells from smokers and non-smokers only significantly differed in a few genes. For example, expression of tumor necrosis factor alpha (*TNF*; fold change: 1.76, p < 0.05) was increased in smokers, while the expression of surface markers, such as CD69 (*CD69*; fold change: 1.7, p < 0.05) and CD56 (*CD56*; fold change: 1.45, p < 0.05) was decreased in smokers. In addition, multiple genes related to cytotoxic NK cell function, such as *CD244*, *NCR1/Nkp46*, and *GZMB* were reduced in smokers, but these changes did not reach statistical significance at p < 0.05 (Table 4).

**Table 3:** Differential Gene Expression of CD56^+^ NLF Cells by Smoking Status.

Gene	Nonsmoker Counts	Smoker Counts	Fold Change	P-Value
*ITGA2*	498.95 ± 243.76	243.56 ± 166.85	2.05	0.022

**Table 4:** Differential Gene Expression of CD56^+^ PB Cells by Smoking Status.

Gene	Nonsmoker Counts	Smoker Counts	Fold Change	P-Value
*CD69*	1736.05 ± 686.92	1021.63 ± 512.55	−1.7	0.022
*NCAM1 (CD56)*	1794.38 ± 342.19	1238.37 ± 436.24	−1.45	0.023
*TNF*	67.33 ± 26.98	118.6 ± 47.22	1.76	0.007
*NCR1 (NKp46)*	577.69 ± 90.36	396.41 ± 168.63	−1.46	0.051
*CD244*	2605.77 ± 455.08	1872 ± 993.42	−1.39	0.1
*GZMB*	16053.57 ± 2830.38	11684.68 ± 4384.95	−1.37	0.087
*SH2D1B*	6969.02 ± 1286.37	5123.02 ± 2230.81	−1.36	0.087
*JAK1*	7055.59 ± 918.35	5300.19 ± 2050.65	−1.33	0.074

Tables 1–4: (1) Genes with significantly greater expression in CD 56^+^ NLF cells compared to PB CD 56^+^ cells. NLF and PB counts are expressed as Mean ± SD. (2) Genes with significantly greater expression in PB CD 56^+^ cells compared to NLF CD 56^+^ cells. NLF and PB counts are expressed as Mean ± SD. (3) Gene differentially regulated by smoking status in NLF CD 56^+^ cells. Nonsmoker and Smoker Counts are expressed as Mean ± SD. (4) Genes differentially regulated by smoking status in PB CD 56^+^ cells. Nonsmoker and Smoker Counts are expressed as Mean ± SD. Shaded rows are values approaching statistical significance.

## Discussion

The nasal passages filter 18,000 to 20,000 liters of air every day, thus constantly exposing the nasal mucosa to millions of microbes and inhaled pollutants, such as cigarette smoke. Consequently, immune cells patrolling the nasal mucosa need to be equipped to orchestrate and execute a multitude of host defense responses against inhaled pathogens and pollutants. To fully understand these immune processes, the innate immune cells within the nasal passages must be functionally defined and differentiated from their peripheral blood counterparts. We have previously shown that leukocytes contained in NLF are predominantly composed of CD45^+^CD3^−^CD56^−^CD16^+^ neutrophils and CD45^+^CD3^−^CD56^+^CD16^+/−^ NK cells^16^. Using a newly developed method for CD56^+^ NK/ILC1 cell enrichment, we were able to conduct experiments allowing us to determine the phenotype and gene expression profile of the resident CD56^+^ NK/ILC1 cell population in the nasal mucosa and compare those data to matched peripheral blood CD56^+^ NK/ILC1 cell in both healthy nonsmokers and smokers. Our data demonstrate that (1) based on surface markers and gene expression profiles NLF and PB CD56^+^ NK/ILC1 cells significantly differ in their phenotype, with NLF CD56^+^ NK/ILC1 cells expressing multiple markers of tissue residency, making their surface marker profile more consistent with ieILC1s, (2) due to the significantly lower expression of markers associated with cytotoxic function, NLF CD56^+^ NK/ILC1 cells are likely more responsible for cytokine signaling and immune system orchestration than their PB circulating cytotoxic counterparts, and (3) smoking only moderately affected gene expression profiles in NLF and PB CD56^+^/NK cells. Together these data indicate that in humans, resident NLF CD56^+^ NK/ILC1 cells phenotypically overlap with ieILC1 and are functionally distinct from PB CD56^+^ NK/ILC1 cells.

NK cells are classically divided into CD56^dim^ and CD56^bright^ and have predominantly been studied in PB and lymphoid tissue. However, there is increasing evidence that distinct populations of CD56^+^ NK/ILC1 cells are present in peripheral tissues. In the lung, CD56^dim^ NK cells have been previously identified and it is thought that lung CD56^+^ NK cells are primarily infiltrated conventional CD56^+^ NK/ILC1 cells that are present temporarily, since these cells lack resident tissue markers such as CD69 and CD103^9^. In contrast, we find a similar CD56^dim^ population in the nose, but also demonstrated that nasal CD56^+^ NK/ILC1 cells are CD69^+^ and CD103^+^, a first indication that these cells are phenotypically distinct and likely functionally different from both lung and PB CD56^+^ NK/ILC1 cells, which similar to previous reports were primarily CD56^dim^CD16^+^ (50–70%) and negative for markers of tissue residency (CD69, CD103)^24^.

Expression of CD69 and CD103 in NLF CD56^+^ NK/ILC1 cells indicates tissue residency and phenotypically distinguishes them from conventional PB CD56^+^ NK/ILC1 cells. More recently expression of CD69 and CD103 in CD56^+^ cells has been suggested to also be associated with ieILC1s^1,25^. How tissue resident NK cells differ from ieILC1s in human tissues is not well understood. To distinguish these two subgroups of cells in various tissues, surface marker expression of NK1.1/CD161, TNFSF10/TRAIL, ITAG2/CD49b, and NKp46 was used to sort these cell types and subsequently analyze their genomic signatures. Liver ILC1s were considered NK1.1/CD161^+^, ITAG2/CD49b^−^, and TNFSF10/TRAIL^+^ and liver NK cells NK1.1/CD161^−^, ITAG2/CD49b^+^, and TNFSF10/TRAIL^−^ and these two cell types showed unique gene expression signatures^26^. Interestingly, genomic expression profiles of intraepithelial lymphocytes from these tissues showed significant overlap with both ILC1s and NK cells^26^, suggesting that ieILC1s and tissue NK cells may not be two distinct cell types. Furthermore, recent studies using mass cytometry including a broad range of surface markers identified ieILC1-like cells that fell within the broader category of NK/ILC1 cells in these tissues^2^, further supporting the notion that the NLF CD56^+^ NK/ILC1 cells identified here significantly overlap with previously described ieILC1s^1–3^.

Based on our gene expression data, we hypothesize that NLF CD56^+^ NK/ILC1 cells have little cytolytic function but are more involved in cytokine expression and orchestration of immune responses^16^. Specifically, NLF CD56^+^ NK/ILC1 cells showed increased expression of genes related to cell signaling and migration (*MAPK3*, *ITGA2*, *IFNGR2*) as well as cytokine production (*TNFSF10/TRAIL* and *IL8*) (Table 2), suggesting an immune orchestrating role. In contrast, PB CD56^+^ NK/ILC1 cells showed increased expression of genes classically found in cytotoxic NK cells (*PRF1*, *GNLY*, *RANTES*, *GZMB*) (Table 3), which were almost absent in NLF CD56^+^ NK/ILC1cells. Thus, as expected based on surface markers and gene expression profiles, PB CD56^+^ NK/ILC1 cells are predominantly cytotoxic and NLF CD56^+^ NK/ILC1 cells align most closely with a more cytokine producing phenotype, which traditionally has been associated with CD56^bright^CD16^−^ NK cell populations^24^. These data also agree with our previous studies demonstrating that PB CD56^+^ NK/ILC1 cells robustly produce Granzyme B and to a lesser extent cytokines, such as IFNγ or IL-4^27^, further supporting a more conventional NK cell phenotype as compared to the ILC1 phenotype which lacks Granzyme B production, but produces IFNγ^3,28^. Furthermore, GSEA analysis also suggested that the PB CD56^+^ NK/ILC1 gene expression was enriched and aligned well with traditional cytotoxic NK cell gene expression, while NLF CD56^+^ NK/ILC1 cells aligned less well with gene expression sets studying cytotoxic NK cell subtypes.

In the nasal mucosa, the NLF CD56^+^ NK/ILC1 cells are likely playing an important role orchestrating immune responses against invading microbes. However, malfunction of tissue associated NK cells or ieILC1s, like the NLF CD56^+^ NK/ILC1 cells described here, has also been associated with several autoimmune or chronic inflammatory diseases^29^, such as CD56^+^CD103^+^ ieILC1s being associated with chronic inflammatory bowl syndrome^3^. In patients with COPD, ILC1 cells are thought to be derived from the local pool of innate lymphoid cells group 2 (ILC2s) and the frequency of circulating ILC1s correlates with markers of disease severity and susceptibility to exacerbations in these patients^30^. In addition, exposure to cigarette smoke recapitulated this shift from ILC2 to ILC1 in the lung^30^, suggesting that smoking enhances the presence of ILC1s in the lung. Interestingly, the only gene that was differentially expressed in our NLF CD56^+^ NK/ILC1cell population was *ITGA2*, which was significantly reduced in smokers. *ITGA2/CD49b* is a pan-NK cell marker and has been used to distinguish NK cells from ILC1s^26^. In addition, in mice, low ITAG2/CD49b expression was associated with reduced cytotoxic function and CD49b expression could therefore be used to identify functionally distinct subsets of NK cells^31^. However, it is unclear whether the reduced *ITGA2/CD49b* expression in NLF CD56^+^ NK/ILC1 cells from human smokers indicates the presence of another unique NK/ILC1 cell subset in these subjects as compared to nonsmokers.

Similar to previous studies, PB CD56^+^ NK/ILC1 cells from smokers showed an increased expression of pro-inflammatory cytokines (TNF), but markers of immune function including cellular migration, activation, signaling, and cytotoxic function were suppressed (*CD69, NCAM1, JAK1, GZMB*, etc. (Table 5)) as compared to nonsmokers^16,32,33^. In addition to increased inflammatory signaling, a suppression of immune function was observed with a loss of NK cell markers, such as CD56 and NKp46 (*NCAM1* and *NCR1*), and a reduction in the cytolytic capacity of NK cells via loss of granzyme b (*GZMB*) expression. A reduction in expression of NKp46 suggests a reduction in NK cell ability to activate cytotoxic responses directed at a target cell, as the NKp46 receptor is essential for viral and bacterial ligands to bind to NK cells^34^. The loss of CD56 (*NCAM1*) also suggests impairment of NK cell cytotoxic function^35^. Thus, while smoking-induced gene expression changes in PB CD56^+^ NK/ILC1 cells are consistent with previous findings, it is unclear how resident NLF CD56^+^ NK/ILC1 cells are affected by chronic cigarette smoke exposure. Our previous studies suggest that smoking reduces the presence of cytotoxic NK cells in the nasal mucosa following a viral infection^16^. Based on the data presented here, it seems likely that these differences are a reflection of smoking-induced changes in PB NK cells and recruitment of these cells into the nasal mucosa following a viral infection.

**Table 5:** Subject Demographics.

	**Nonsmoker**	**Smoker**
BMI	26.32 ± 7.13	30.16 ± 6.22
Age	26.89 ± 6.85	36.67 ± 8.05*
Sex: female/male	5/4	5/4
Race: White/African American/Asian	5/2/2	6/3/0
Serum Cotinine (ng/nM protein)	2.15 ± 1.19	1649.50 ± 836.01*

Values are Mean ± SE

Table 5: Subject demographic data. Subject BMI, age, and serum cotinine are shown as Mean ± SE. Comparisons were made between nonsmokers and smokers, *P ≤ 0.05.

Overall, this manuscript provides the first characterization of a resident NLF CD56^+^ NK/ILC1 cell population in humans. The (CD56^dim^CD16^dim^CD69^+^CD103^+^) population is both phenotypically and functionally distinct from its circulating peripheral blood counterpart (CD56^dim^CD16^bright^CD69^−^CD103). Based on data presented here we suggest that NLF CD56^+^ NK/ILC1 cells overlap phenotypically with ieILC1s and are primarily immune orchestrating, while PB CD56^+^ NK/ILC1 cells act as non-antigen-restricted cytotoxic lymphocytes. We also expand previous findings in that the gene expression profiles and potential function of CD56^+^ NK/ILC1 cells within these two compartments can be altered by cigarette smoke exposure, with more prominent differences seen in PB CD56^+^ NK/ILC1 cells. Together the data shown here characterize NLF (CD56^dim^CD16^dim^CD69^+^CD103^+^) cells as a novel NK/ILC1-like cell population with a gene expression profile and surface marker presentation consistent with tissue residency and potential to play a significant role in host-defense and overall immune orchestration in normal healthy individuals.

## Materials and Methods

### Subject Recruitment

Healthy adult subjects aged 21–47 years old were recruited. The exclusion criteria for this study were: current symptoms of allergic rhinitis, asthma, forced expiratory volume 1 less than 75% predicted at screen, chronic obstructive pulmonary disorder, cardiac disease, any chronic cardiorespiratory condition, bleeding disorders, recent nasal surgery, immunodeficiency, or current pregnancy. At the study visit informed consent was obtained from all subjects for study participation and demographic information, pregnancy tests (for female subjects), vital signs, nasal lavage fluid (NLF), blood, and urine were collected. Both cigarette smokers (n = 9) and non-smokers (n = 9) were recruited for the study. Smokers self-identified and had a smoking history of greater than 5 cigarettes per week. Smoking status was confirmed by assessing urine cotinine levels, which were significantly higher in smokers than non-smokers. There were no significant differences in body mass index (BMI), sex, and ethnicity between treatment groups (Table 5). Smokers were slightly, but significantly older than non-smokers (smokers: 36.67 ± 8.05 and non-smokers 26.89 ± 6.85).

The protocol was submitted to and approved by the University of North Carolina at Chapel Hill Biomedical Institutional Review Board and all methods were performed in accordance with relevant guidelines and regulations.

### NLF Collection and NK Cell Enrichment

NLF was obtained as in our previous studies^16,21,22,32,36,37^. Briefly, nostrils were repeatedly sprayed with 0.9% sterile, normal saline irrigation solution (a total of 4 ml per nostril) and expelled into collection cups. The cellular component was then isolated from the NLF using filtration (40 μm cell strainer) and centrifugation. The cell pellets were then either stained for flow cytometric analysis or enriched for NK cells utilizing a combination of two antibody-based selection cocktails: NLF cells were treated with a neutrophil positive selection antibody cocktail (anti-human CD66abce-biotin, Miltenyi Biotec Inc., San Diego, CA, USA), as well as a CD56^+^/NK cell negative selection antibody cocktail (biotinylated monoclonal anti-human antibody cocktail, Cell ThermoFisher Scientific, Waltham, MA, USA), washed with Dulbecco’s phosphate buffered saline (DPBS) (GIBCO/Thermo Fischer, Waltham, MD), and plated in triplicate wells on a 96-well high binding plate coated with a molar excess of streptavidin (Sigma-Aldrich, St. Louis, MO) as cell loss was too great using the kit provided streptavidin coated magnetic beads. After incubation at room temperature for 20 minutes, the non-attaching enriched CD56^+^/NK cells were carefully removed and were either stained for flow cytometric analysis or re-suspended in a commercially available lysis buffer (RLT, QIAGEN Sciences, Germantown, MD, USA) for gene expression analysis.

### Isolation of NK Cells from Peripheral Blood

Venous whole blood was collected into vacutainer tubes containing heparin (BD, Franklin Lakes, New Jersey). Blood was diluted 1:4 with DPBS and centrifuged over Lymphoprep density gradient medium (STEMCELL Technologies Inc., Vancouver, BC, Canada) according to manufacturer’s instructions to fractionate whole blood components. PB mononuclear cell fractions were collected and incubated with a biotinylated NK cell negative selection antibody cocktail, as above. Cells were combined with streptavidin-coated magnetic beads from the selection kit above and subjected to magnetic separation to create an NK cell enriched supernatant. The resulting enriched NK cells were either stained for flow cytometric analysis or re-suspended in a commercially available lysis buffer for gene expression analysis, as above. It should be noted that enrichment methods for NLF and PB cells differed slightly. Specifically, the use of a streptavidin coated plates was used for NLF enrichment rather than the kit provided streptavidin coated beads, which was used for PB cells. This deviation from the kit protocol was devised in response to the small starting number of NLF NK cells and the large percentage of NK cells lost to the bead column.

### NK Cell Enrichment Validation

NLF cells and enriched CD56^+^NK/ILC1 cells were stained with an NK cell identifying antibody cocktail (human CD45, CD56, and CD16) (Supplementary Table 1), fixed in 0.5% paraformaldehyde, and analyzed on a LSR II flow cytometer (BD, Franklin Lakes, NJ) (Fig. 1). Cell viability was assessed via a membrane integrity-based viability assay compatible with cell fixation (LIVE/DEAD® Fixable Dead Cell Stains, ThermoFisher, Waltham, MA).

### Surface Marker Phenotyping of CD56+ NK/ILC1 Cells in NLF and PB

To compare surface marker expression between NLF and PB cells, PB CD56^+^ NK/ILC1 cells were first labeled with Carboxyfluorescein succinimidyl ester (CFSE) (Cayman Chemical, Ann Arbor, MI). Equal numbers of PB and NLF CD56^+^ NK/ILC1 cells from the same subject were subsequently stained for human CD45, CD56, CD16, CD69, and CD103 (Supplementary Table 1). Stained cell pellets from PB and NLF from the same subjects were combined, fixed in 0.5% paraformaldehyde, and acquired on a LSR II flow cytometer (Fig. 2).

### Nanostring Based Gene Expression

**Figure 3:**
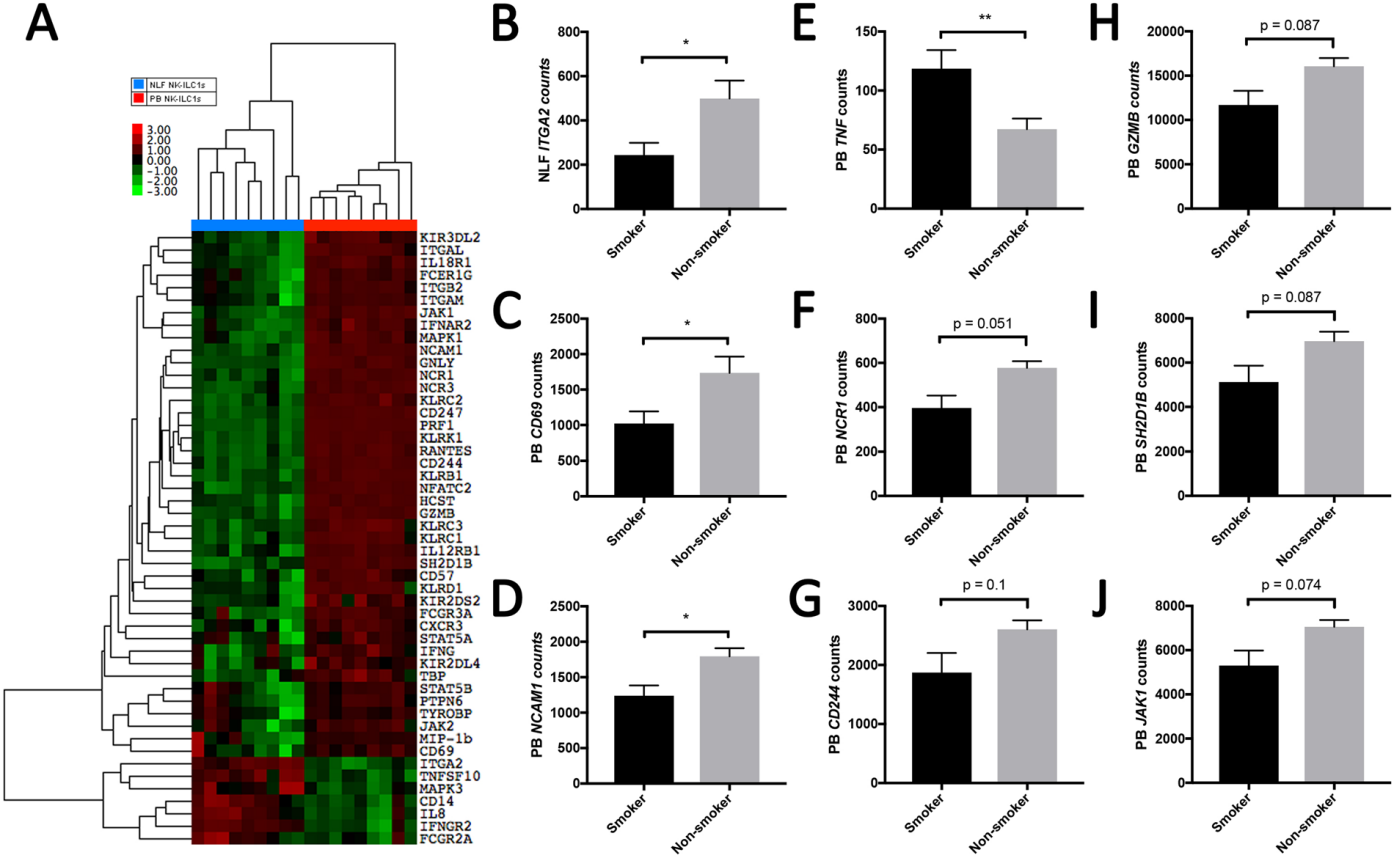
Compartmental differential gene expression, NLF versus PB CD56^+^ cells. (**A**) Heat map of differential gene expression in NLF (blue bar) versus peripheral blood-derived (red bar) NK/ILC1 cells, red = upregulation, green = downregulation. (**B**) Bar graph of differential *ITGA2* expression in NLF CD56^+^ NK/ILC1s in smokers (black bar) and nonsmokers (grey bar). (**C–J**) Bar graphs of differential gene expression in PB CD56^+^ NK/ILC1s in smokers (black bar) and nonsmokers (grey bar): (**C**) *CD69*, (**D**) *NCAM1*, (**E**) *TNF*, (**F**) *NCR1*, (**G**) *CD244*, (**H**) *GZMB*, (**I**) *SH2D1B*, (**J**) *JAK1*. Mean ± SE, *P ≤ 0.05, **P ≤ 0.01.

A 71-gene Nanostring (Nanostring, Seattle, WA) nCounter® code set was custom designed (Supplementary Table 2) to evaluate NK cell activation and effector related gene expression. Genes of interest were chosen based on the KEGG NK cell activation pathway and housekeeping genes were selected based on previously published data in peripheral blood mononuclear cells^38^. The custom nCounter® code set was used to analyze gene expression profiles in CD56^+^ cells from PB and NLF (Fig. 3). Nanostring technology was chosen, as opposed to other commercially available methodologies, to avoid amplification bias, its effectiveness in analysis of small tissue samples, and the ability to complete the analysis using whole cell lysate. In addition, the Nanostring technology facilitated the custom gene array design, specifically analyzing NK cell-specific gene expression. More specifically, the number of cells and the amount of genetic material available from the NLF compartment severely limited our ability to perform comprehensive analysis of gene expression. Therefore, a platform allowing robust and cell-type specific analysis of small amounts of genetic material was chosen^39^.

### Statistical Analysis

Nanostring gene expression data were normalized to positive and negative control genes and to the geometric mean of stable housekeeping genes (POLR2A and LDHA) and analyzed using the nSolver™ software provided by the manufacturer. Genes with expression levels below the geometric mean count of the negative controls plus two standard deviations (counts of 17.5 or less) were excluded from analysis.

To better understand the contribution to variance and determine the appropriate model for analysis, a random forest analysis was conducted on the measured covariates within the data set (R, Vienna, Austria; https://cran.r-project.org/web/packages/rfPermute/rfPermute.pdf)^40,41^. This analysis suggested that the variation within the data set was not attributable to the covariates, including age (p = 0.63). As a secondary analysis, regression analysis was performed and it was demonstrated that age does not have a significant impact on the gene expression for all except IFNG (p = 0.03) in the PB CD56^+^ NK/ILC1. These data are shown in Supplementary Table 5. Additionally, age has not been shown to be predictive of smoking in previous studies^16,21,22,32^. Based upon these analysis, a two tailed t-test was used to analyze gene expression for differences between smokers and non-smokers.

In all gene expression analyses, to control for multiple comparisons, we used a false discovery correction with a Q value of 10%^42^. Consequently, the uncorrected p-value of ≤0.05 was adjusted to a more stringent p-value of ≤0.027 to control for multiple comparisons. In addition, a fold change of greater than 1.5 was used with the adjusted p-value to determine significance. Cutoff criteria for significance was based on MicroArray Quality Control (MAQC) project findings and the Guo *et al*. suggestion that using both fold change and p-value cutoff showed increased reproducibility across gene expression platforms and reliable deduction of biological impact^43–46^. Individual genes were compared between compartments (i.e. NLF versus PB) and between smoking statuses using a two-tailed T-test. Heat maps were generated using Spearman correlation and median distance linkage.

Flow cytometry post-acquisition analyses were performed with FlowJo v.8 (FlowJo, LLC, Ashland, OR). Subject demographic data were analyzed using student t-tests in GraphPad Prism 6 (GraphPad, La Jolla, CA) and R as above.

### Pathway analysis

Pathway analysis was completed using Gene set enrichment analysis (GSEA) on the differentially expressed genes in both the NLF and PB (Broad Institute, Inc., Massachusetts Institute of Technology, and Regents of the University of California)^47,48^. The top 50 overlaps were computed with FDR q-value below 0.05 using Molecular Signatures Database v6.1, collections 2, 5, and 7 (Supplementary Tables 3 and 4).

### Data availability

The datasets generated and analyzed during the current study are available from the corresponding author upon reasonable request.

### Disclaimer

Research reported in this publication was in part supported by NIH and the FDA Center for Tobacco Products (CTP). The content is solely the responsibility of the authors and does not necessarily represent the official views of the National Institutes of Health or the Food and Drug Administration.

## Electronic supplementary material

Supplementary Materials
