# Supplementary material for: Distinguishing Human Peripheral Blood NK Cells from CD56dimCD16dimCD69+CD103+ Resident Nasal Mucosal Lavage Fluid Cells
Source: Sci Rep. 2018 Feb 21;8:3394. doi: 10.1038/s41598-018-21443-5 (PMC5821812; doi:10.1038/s41598-018-21443-5)
Supplement: Supplementary file 1 — Supplementary Materials [file 41598_2018_21443_MOESM1_ESM.pdf]

Distinguishing Human Peripheral Blood NK Cells from CD56^{dim}CD16^{dim}CD69^{+}CD103^{+} Resident Nasal Mucosal Lavage Fluid Cells

Meghan E. Rebuli[1] Erica A. Pawlak[2], Dana Walsh[1], Elizabeth M. Martin[3], and Ilona Jaspers*[1,2,4]

Supplementary Table 1: Antibodies

Epitope	Fluorochrome	Isotype	Manufacturer
-	CFSE	-	BD
CD 16	PerCP-Cy5.5	IgG1	BioLegend
CD 45	APC-Cy7	IgG1	BioLegend
CD 56	PE	IgG1	BD
CD 69	Brilliant Violet 421	IgG1	BioLegend
CD 103	APC	IgG1	BioLegend

Supplementary Table 2: Genes in Nanostring Custom Code Set

Gene Name	Accession Number	Target Region	Target Sequence
CD14	NM_000591.2	886-985	GCCCAAGCACACTCGCCTGCCTTTTCCTGCGAACAGGTTCGCGCCTTCCC GGCCCTTACCAGCCTAGACCTGTCTGACAATCCTGGACTGGGCGAACGCG
CD244	NM_016382.2	1151-1250	AAGAGGAACCACAGCCCTTCCTTCAATAGCACTATCTATGAAGTGATTGGAA AGAGTCAACCTAAAGCCCAGAACCCTGCTCGATTGAGCCGCAAAGAGC
CD247	NM_198053.1	1491-1590	TGGCAGGACAGGAAAAACCCGTCAATGTACTAGGATACTGCTGCGTCATTA CAGGGCACAGGCCATGGATGGAAAACGCTCTCTGCTCTGCTTTTTTTCT
CD57	NM_018644.3	2389-2488	GAGGGAGGCCTGAGCACACTGCTTTGGAAATTATTCTAAACACAAAAAAGG GAAAGAAAATGTTATTTCTCCCTAAGTCAGGAGCATGCAGAGCTAGCCC
CD69	NM_001781.1	461-560	AGGACATGAACTTTCTAAAACGATACGCAGGTAGAGAGGAACACTGGGTTG GACTGAAAAAGGAACCTGGTCACCCATGGAAGTGGTCAAATGGCAAAGA
CSF2	NM_000758.2	476-575	AGATGAGGCTGGCCAAGCCGGGGAGCTGCTCTCTCATGAAACAAGAGCTA GAAACTCAGGATGGTCATCTTGGAGGGACCAAGGGGTGGGCCACAGCCAT
CXCL10	NM_001565.1	41-140	GCAGAGGAACCTCCAGTCTCAGCACCATGAATCAAACTGCGATTCTGATTT GCTGCCTTATCTTTCTGACTCTAAGTGGCATTCAAGGAGTACCTCTCTC
CXCR3	NM_001504.1	81-180	GTGAGTGACCACCAAGTGCTAAATGACGCCGAGGTTGCCGCCCTCCTGGA GAACTTCAGCTCTTCCTATGACTATGGAGAAAACGAGAGTGACTCGTGCT
FASLG	NM_000639.1	626-725	TCCATGCCTCTGGAATGGGAAGACACCTATGGAATTGTCCTGCTTTCTGGA GTGAAGTATAAGAAGGGTGGCCTTGTGATCAATGAAACTGGGCTGTACT
FCER1G	NM_004106.1	37-136	AGTGGTCTTGCTCTTACTCCTTTTGGTTGAACAAGCAGCGGCCCTGGGAGA GCCTCAGCTCTGCTATATCCTGGATGCCATCCTGTTTCTGTATGGAATT
FCGR2A	NM_021642.2	1-100	GTCTCTTAAAACCCACTGGACGTTGGCACAGTGCTGGGATGACTATGGAGA CCCAAATGTCTCAGAATGTATGTCCCAGAAACCTGTGGCTGCTTCAACC
FCGR2B	NM_001002273 .1	871-970	AGGCTGACAAAGTTGGGGCTGAGAACACAATCACCTATTCACTTCTCATGC ACCCGGATGCTCTGGAAGAGCCTGATGACCAGAACCGTATTTAGTCTCC

Gene Name	Accession Number	Target Region	Target Sequence
FCGR3A	NM_000569.6	1645-1744	AAATCATGAGGGTGACGTAGAATTGAGTCTTCCAGGGGACTCTATCAGAAC TGGACCATCTCCAAGTATATAACGATGAGTCCTCTTAATGCTAGGAGTA
GNLY	NM_006433.2	306-405	CAGGAGCTGGGCCGTGACTACAGGACCTGTCTGACGATAGTCCAAAAACT GAAGAAGATGGTGGATAAGCCCACCCAGAGAAGTGTTTCCAATGCTGCGA
GZMB	NM_004131.3	541-640	ACACTACAAGAGGTGAAGATGACAGTGCAGGAAGATCGAAAGTGCGAATCT GACTTACGCCATTATTACGACAGTACCATTGAGTTGTGCGTGGGGGACC
HCST	NM_001007469 .1	133-232	ATCCTCTTCCTGCTTTTGCTCCCAGTGGCTGCAGCTCAGACGACTCCAGGA GAGAGATCATCACTCCCTGCCTTTTACCCTGGCACTTCAGGCTCTTGTT
IFNAR1	NM_000629.2	3124-3223	CTAATCAGCTCTCAGTGATCAACCCACTCTTGTTATGGGTGGTCTCTGTCAC TTTGAATGCCAGGCTGGCTTCTCGTCTAGCAGTATTCAGATACCCCTT
IFNAR2	NM_000874.3	632-731	AAATACCACAAGATCATTTTGTGACCTCACAGATGAGTGGAGAAGCACACA CGAGGCCTATGTCACCGTCCTAGAAGGATTCAGCGGGAACACAACGTTG
IFNG	NM_000619.2	971-1070	ATACTATCCAGTTACTGCCGGTTTGAAAATATGCCTGCAATCTGAGCCAGTG CTTTAATGGCATGTCAGACAGAACTTGAATGTGTCAGGTGACCCTGAT
IFNGR1	NM_000416.1	1141-1240	CCCGGGCAGCCATCTGACTCCAATAGAGAGAGAGAGTTCTTCACCTTTAAG TAGTAACCAGTCTGAACCTGGCAGCATCGCTTTAAACTCGTATCACTCC
IFNGR2	NM_005534.3	800-899	CAGTGGCCCTGAGCAATAGCACGAGGCCTGTTGTCTACCAAGTGCAGTTTA AATACACCGACAGTAAATGGTTCACGGCCGACATCATGTCCATAGGGGT
IL12RB1	NM_005535.1	449-548	GTGACCCTGCAGCTCTACAACTCAGTTAAATATGAGCCTCCTCTGGGAGAC ATCAAGGTGTCCAAGTTGGCCGGGCAGCTGCGTATGGAGTGGGAGACCC
IL13	NM_002188.2	517-616	TTTCTTTCTGATGTCAAAAATGTCTTGGGTAGGCGGGAAGGAGGGTTAGGG AGGGGTAAAATTCCTTAGCTTAGACCTCAGCCTGTGCTGCCCGTCTTCA
IL17A	NM_002190.2	241-340	TACTACAACCGATCCACCTCACCTTGGAATCTCCACCGCAATGAGGACCCT GAGAGATATCCCTCTGTGATCTGGGAGGCAAAGTGCCGCCACTTGGGCT
IL18R1	NM_003855.2	2026-2125	GAATGAGGGGATTTTAAGTGTCTGAAGAGGCATTTTCTAGGGACCAGTGGG TGACTGAGTAACTGAAATGCTGCTTTCACTCCCTAACACCATGGATCTG
IL22	NM_020525.4	320-419	CTATCTGATGAAGCAGGTGCTGAACTTCACCCTTGAAGAAGTGCTGTTCCC TCAATCTGATAGGTTCCAGCCTTATATGCAGGAGGTGGTGCCCTTCCTG
IL4	NM_000589.2	626-725	GACACTCGCTGCCTGGGTGCGACTGCACAGCAGTTCCACAGGCACAAGCA GCTGATCCGATTCCTGAAACGGCTCGACAGGAACCTCTGGGGCCTGGCGG
IL5	NM_000879.2	106-205	CCACAGAAATTCCCACAAGTGCATTGGTGAAAGAGACCTTGGCACTGCTTT CTACTCATCGAACTCTGCTGATAGCCAATGAGACTCTGAGGATTCCTGT
IL8	NM_000584.2	26-125	ACAGCAGAGCACACAAGCTTCTAGGACAAGAGCCAGGAAGAAACCACCGG AAGGAACCATCTCACTGTGTGTAAACATGACTTCCAAGCTGGCCGTGGCT
ITGA2	NM_002203.2	476-575	CAACGGGTGTGTGTTCTGACATCAGTCCTGATTTTCAGCTCTCAGCCAGCT TCTCACCTGCAACTCAGCCCTGCCCTTCCCTCATAGATGTTGTGGTTGT
ITGAL	NM_002209.2	3906-4005	GTGAGGGCTTGTCATTACCAGACGGTTCACCAGCCTCTCTTGGTTTCCTTC CTTGGAAGAGAATGTCTGATCTAAATGTGGAGAAACTGTAGTCTCAGGA
ITGAM	NM_000632.3	516-615	GCCCTCCGAGGGTGTCCTCAAGAGGATAGTGACATTGCCTTCTTGATTGAT GGCTCTGGTAGCATCATCCCACATGACTTTCGGCGGATGAAGGAGTTTG
ITGAX	NM_000887.3	701-800	CCCCTCAGCCTGTTGGCTTCTGTTCACCAGCTGCAAGGGTTTACATACACG GCCACCGCCATCCAAAATGTCGTGCACCGATTGTTCCATGCCTCATATG
ITGB2	NM_000211.2	521-620	CATCGACCTGTACTATCTGATGGACCTCTCCTACTCCATGCTTGATGACCTC AGGAATGTCAAGAAGCTAGGTGGCGACCTGCTCCGGGCCCTCAACGAG
JAK1	NM_002227.1	286-385	GAGAACACCAAGCTCTGGTATGCTCCAAATCGCACCATCACCGTTGATGAC AAGATGTCCCTCCGGCTCCACTACCGGATGAGGTTCTATTTCACCAATT

Gene Name	Accession Number	Target Region	Target Sequence
JAK2	NM_004972.2	456-555	CTCCTCCCGCGACGGCAAATGTTCTGAAAAAGACTCTGCATGGGAATGGCC TGCCTTACGATGACAGAAATGGAGGGAACATCCACCTCTTCTATATATC
KIR2DL2	NM_014219.2	815-914	TCTCCTTCATCGCTGGTGCTCCAACAAAAAAAATGCTGCGGTAATGGACCA AGAGTCTGCAGGGAACAGAACAGCGAATAGCGAGGACTCTGATGAACAA
KIR2DL4	NM_002255.5	16-115	GCGTCCTGGCAGCAGAAGCTGCACCATGTCCATGTCACCCACGGTCATCAT CCTGGCATGTCTTGGGTTCTTCTTGGACCAGAGTGTGTGGGCACACGTG
KIR2DS2	NM_012312.2	857-956	CAAGAGCCTGCAGGGAACAGAACAGTGAACAGCGAGGATTCTGATGAACA AGACCATCAGGAGGTGTCATACGCATAATTGGATCACTGTGTTTTCACAC
KIR3DL2	NM_006737.2	885-984	TGCCACCCACGGAGGGACCTACAGATGCTTCGGCTCTTTCCGTGCCCTGC CCTGCGTGTGGTCAAACTCAAGTGACCCACTGCTTGTTTCTGTCACAGGA
KLRB1	NM_002258.2	86-185	TGAGTTAAACTTACCCACAGACTCAGGCCCAGAAAGTTCTTCACCTTCATCT CTTCCTCGGGATGTCTGTCAGGGTTCACCTTGGCATCAATTTGCCCTG
KLRC1	NM_002259.3	336-435	ACCTATCACTGCAAAGATTTACCATCAGCTCCAGAGAAGCTCATTGTTGGGA TCCTGGGAATTATCTGTCTTATCTTAATGGCCTCTGTGGTAACGATAG
KLRC2	NM_002260.3	943-1042	TATGTGAGTCAGCTTATAGGAAGTACCAAGAACAGTCAAACCCATGGAGAC AGAAAGTAGAATAGTGGTTGCCAATGTCTCAGGGAGGTTGAAATAGGAG
KLRC3	NM_007333.2	599-698	GGGTGACAATAAATGGTTTGGCTTTCAAACATGAGATAAAAGACTCAGATCA TGCTGAACGTAACTGTGCAATGCTACATGTACGTGGACTTATATCAGA
KLRD1	NM_007334.2	216-315	TACACATCGTGCCTTCTCTACTTCGCTCTTGGAACATAATTTCTCATGGCAG CTTTTACTAAACTGAGTATTGAGCCAGCATTTACTCCAGGACCCAACA
KLRK1	NM_007360.1	761-860	GGACCAGGATTTACTTAAACTGGTGAAGTCATATCATTGGATGGGACTAGTA CACATTCCAACAAATGGATCTTGGCAGTGGGAAGATGGCTCCATTCTC
LDHA	NM_005566.1	986-1085	CAGAATGGAATCTCAGACCTTGTGAAGGTGACTCTGACTTCTGAGGAAGAG GCCCGTTTGAAGAAGAGTGCAGATACACTTTGGGGGATCCAAAAGGAGC
MAPK1	NM_138957.2	431-530	ACTGCCAGAGAACCCTGAGGGAGATAAAAATCTTACTGCGCTTCAGACATG AGAACATCATTGGAATCAATGACATTATTCGAGCACCAACCATCGAGCA
MAPK3	NM_001040056 .1	581-680	AACGTGCTCCACCGAGATCTAAAGCCCTCCAACCTGCTCATCAACACCACC TGCGACCTTAAGATTTGTGATTTCGGCCTGGCCCGGATTGCCGATCCTG
MIP-1a	NM_002983.2	682-781	CTGTGTAGGCAGTCATGGCACCAAAGCCACCAGACTGACAAATGTGTATCG GATGCTTTTGTTCAGGGCTGTGATCGGCCTGGGGAAATAATAAAGATGC
MIP-1b	NM_002984.2	36-135	TTCTGCAGCCTCACCTCTGAGAAAACCTCTTTGCCACCAATACCATGAAGCT CTGCGTGACTGTCCTGTCTCTCCTCATGCTAGTAGCTGCCTTCTGCTC
NCAM1	NM_000615.5	1621-1720	GGTATTTGCCTATCCCAGTGCCACGATCTCATGGTTTCGGGATGGCCAGCT GCTGCCAAGCTCCAATTACAGCAATATCAAGATCTACAACACCCCCTCT
NCR1	NM_001242357 .1	707-806	GAGGGAGTCACTGCAGGGAAAGAGGGACACTGGCATTCCATTTGTCAGAG CATCCCGGACGATGCAGAGGGTGGGAGAACTACATGCTAAATTTCTTTTT
NCR2	NM_004828.3	799-898	CTTCAACAGGTCACGGACCTTCCCTGGACCTCAGTTTCCTCACCTGTAGAG AGAGAAATATTATATCACACTGTTGCAAGGACTAAGATAAGCGATGATG
NCR3	NM_147130.2	656-755	ATCCTCAGCTAGGGGCTGGTACAGTCCTCCTCCTTCGGGCTGGATTCTATG CTGTCAGCTTTCTCTCTGTGGCCGTGGGCAGCACCGTCTATTACCAGGG
NFATC2	NM_012340.3	1816-1915	GACGGACATTGGAAGAAAGAACACGCGGGTGAGACTGGTTTTCCGAGTTC ACATCCCAGAGTCCAGTGGCAGAATCGTCTCTTTACAGACTGCATCTAAC
NR0B2	NM_021969.1	736-835	TCTTTCGCCCTATCATTGGAGATGTTGACATCGCTGGCCTTCTTGGGGACA TGCTTTTGCTCAGGTGACCTGTTCCAGCCCAGGCAGAGATCAGGTGGGC
POLR2A	NM_000937.2	3776-3875	TTCCAAGAAGCCAAAGACTCCTTCGCTTACTGTCTTCCTGTTGGGCCAGTC CGCTCGAGATGCTGAGAGAGCCAAGGATATTCTGTGCCGTCTGGAGCAT

Gene Name	Accession Number	Target Region	Target Sequence
PRF1	NM_005041.3	2121-2220	ACTGTTTTTCAGGGAGGTGGCTGGGTTTACACGCTAATCCCGATTCACCCT GTCCAAACTGCCTAAGCCCTCCGCCATTCTCAAGCCCTGCAGTCACAGC
PTPN11	NM_002834.3	1481-1580	TGTCAAATACTGGCCTGATGAGTATGCTCTAAAAGAATATGGCGTCATGCGT GTTAGGAACGTCAAAGAAAGCGCCGCTCATGACTATACGCTAAGAGAA
PTPN6	NM_002831.5	1735-1834	TGGTGCAGACGGAGGCGCAGTACAAGTTCATCTACGTGGCCATCGCCCAG TTCATTGAAACCACTAAGAAGAAGCTGGAGGTCCTGCAGTCGCAGAAGGG
RANTES	NM_002985.2	281-380	AGTGTGTGCCAACCCAGAGAAGAAATGGGTTCGGGAGTACATCAACTCTTT GGAGATGAGCTAGGATGGAGAGTCCTTGAACCTGAACTTACACAAATTT
SDHA	NM_004168.1	231-330	TGGAGGGGCAGGCTTGCGAGCTGCATTTGGCCTTTCTGAGGCAGGGTTTA ATACAGCATGTGTTACCAAGCTGTTTCCTACCAGGTCACACACTGTTGCA
SH2D1B	NM_053282.4	546-645	GTTGAAGAGATGAGTAACAGTTCTCACTGATGACCCACTTCTGCAGGCATA GGTCCAGAGCACCAAACTCTAGTGGACAATTCAGACTCTCCTGGTTGTG
STAT1	NM_007315.2	206-305	TTTGCTGTATGCCATCCTCGAGAGCTGTCTAGGTTAACGTTCGCACTCTGT GTATATAACCTCGACAGTCTTGGCACCTAACGTGCTGTGCGTAGCTGCT
STAT5A	NM_003152.2	3461-3560	GAGACAGAGAGAGAGAAAGAGAGAGTGTGTGGGTCTATGTAAATGCATCTG TCCTCATGTGTTGATGTAACCGATTCATCTCTCAGAAGGGAGGCTGGGG
STAT5B	NM_012448.3	201-300	AAGGAGAAGCCCTTCATCAGATGCAAGCGTTATATGGCCAGCATTTTCCCA TTGAGGTGCGGCATTATTTATCCCAGTGGATTGAAAGCCAAGCATGGGA
TBP	NM_003194.3	26-125	CGCCGGCTGTTTAACTTCGCTTCCGCTGGCCCATAGTGATCTTTGCAGTGA CCCAGCAGCATCACTGTTTCTTGGCGTGTGAAGATAACCCAAGGAATTG
TNF	NM_000594.2	1011-1110	AGCAACAAGACCACCACTTCGAAACCTGGGATTCAGGAATGTGTGGCCTGC ACAGTGAAGTGCTGGCAACCACTAAGAATTCAAACTGGGGCCTCCAGAA
TNFSF10	NM_003810.2	116-215	GGGGGGACCCAGCCTGGGACAGACCTGCGTGCTGATCGTGATCTTCACAG TGCTCCTGCAGTCTCTCTGTGTGGCTGTAACTTACGTGTACTTTACCAAC
TYROBP	NM_003332.2	458-557	CTGCACCTCATTCCAACTCCTACCGCGATACAGACCCACAGAGTGCCATCC CTGAGAGACCAGACCGCTCCCCAATACTCTCCTAAAATAAACATGAAGC

Supplementary Table 2: Accession numbers and target sequences utilized in synthesizing capture and detection probes for NanoString nCounter® custom code set

Supplementary Table 3: PB GSEA

Overlap Results

Collection(s):	C2, C5, C7
# overlaps shown:	50
# genesets in collections:	15527
# genes in comparison (n):	44
# genes in universe (N):	45956

Gene Set Name	# Genes in Gene Set (K)	# Genes in Overlap (k)	k/K	p-value	FDR q-value
KEGG_NATURAL_KILLER_CELL_MEDIATED_CYTOTOXICITY	137	28	0.2044	4.00E-61	6.22E-57
GO_REGULATION_OF_IMMUNE_RESPONSE	858	30	0.035	7.40E-42	5.74E-38
GO_REGULATION_OF_IMMUNE_SYSTEM_PROCESS	1403	32	0.0228	3.40E-39	1.76E-35
GO_IMMUNE_SYSTEM_PROCESS	1984	32	0.0161	2.12E-34	8.24E-31
GO_DEFENSE_RESPONSE	1231	28	0.0227	1.96E-33	6.09E-30
REACTOME_IMMUNOREGULATORY_INTERACTIONS_BETWE	70	13	0.1857	3.64E-27	9.41E-24
GO_IMMUNE_RESPONSE	1100	23	0.0209	5.21E-26	1.15E-22
REACTOME_IMMUNE_SYSTEM	933	22	0.0236	6.31E-26	1.22E-22
GSE45739_UNSTIM_VS_ACD3_ACD28_STIM_NRAS_KO_CD4_	200	14	0.07	5.64E-23	9.73E-20
GSE3565_CTRL_VS_LPS_INJECTED_DUSP1_KO_SPLENOCY	177	13	0.0734	1.23E-21	1.90E-18
GO_SIGNAL_TRANSDUCER_ACTIVITY	1731	23	0.0133	1.44E-21	2.03E-18
GO_REGULATION_OF_INNATE_IMMUNE_RESPONSE	357	15	0.042	3.16E-21	4.09E-18
GO_INNATE_IMMUNE_RESPONSE	619	17	0.0275	6.23E-21	7.44E-18
GO_CELL_ACTIVATION	568	16	0.0282	7.28E-20	8.08E-17
GSE2935_UV_INACTIVATED_VS_LIVE_SENDAI_VIRUS_INF_M	173	12	0.0694	1.04E-19	1.08E-16
GO_REGULATION_OF_DEFENSE_RESPONSE	759	17	0.0224	1.92E-19	1.86E-16
GO_IMMUNE_EFFECTOR_PROCESS	486	15	0.0309	3.24E-19	2.96E-16
CHAN_INTERFERON_PRODUCING_DENDRITIC_CELL	12	7	0.5833	3.52E-19	2.96E-16
GO_RECEPTOR_ACTIVITY	1649	21	0.0127	3.62E-19	2.96E-16
GO_POSITIVE_REGULATION_OF_RESPONSE_TO_STIMULUS	1929	22	0.0114	3.91E-19	3.03E-16
GSE9650_NAIVE_VS_EFF_CD8_TCELL_DN	200	12	0.06	6.17E-19	4.56E-16
GO_POSITIVE_REGULATION_OF_IMMUNE_SYSTEM_PROCES	867	17	0.0196	1.77E-18	1.25E-15
JAATINEN_HEMATOPOIETIC_STEM_CELL_DN	226	12	0.0531	2.74E-18	1.85E-15
GO_POSITIVE_REGULATION_OF_IMMUNE_RESPONSE	563	15	0.0266	2.90E-18	1.87E-15
GSE3565_DUSP1_VS_WT_SPLENOCYTES_UP	163	11	0.0675	5.50E-18	3.42E-15
GO_CELL_SURFACE	757	16	0.0211	6.82E-18	4.07E-15
GSE7509_UNSTIM_VS_FCGRIIB_STIM_DC_DN	171	11	0.0643	9.42E-18	5.42E-15
GSE3565_CTRL_VS_LPS_INJECTED_SPLENOCYTES_UP	173	11	0.0636	1.07E-17	5.95E-15
HAHTOLA_SEZARY_SYNDROM_DN	42	8	0.1905	4.14E-17	2.14E-14
KEGG_GRAFT_VERSUS_HOST_DISEASE	42	8	0.1905	4.14E-17	2.14E-14
GSE22886_NAIVE_CD8_TCELL_VS_MONOCYTE_UP	200	11	0.055	5.43E-17	2.56E-14
GSE45739_UNSTIM_VS_ACD3_ACD28_STIM_WT_CD4_TCELL	200	11	0.055	5.43E-17	2.56E-14
KAECH_NAIVE_VS_DAY15_EFF_CD8_TCELL_DN	200	11	0.055	5.43E-17	2.56E-14
REACTOME_ADAPTIVE_IMMUNE_SYSTEM	539	14	0.026	6.57E-17	3.00E-14
GO_LEUKOCYTE_ACTIVATION	414	13	0.0314	8.60E-17	3.81E-14
GO_POSITIVE_REGULATION_OF_CELL_ACTIVATION	311	12	0.0386	1.30E-16	5.55E-14
GO_SIDE_OF_MEMBRANE	428	13	0.0304	1.32E-16	5.55E-14
GO_SIGNALING_RECEPTOR_ACTIVITY	1393	18	0.0129	2.04E-16	8.35E-14
GO_EXTERNAL_SIDE_OF_PLASMA_MEMBRANE	238	11	0.0462	3.76E-16	1.50E-13
GO_REGULATION_OF_RESPONSE_TO_STRESS	1468	18	0.0123	5.07E-16	1.97E-13
GO_CELLULAR_DEFENSE_RESPONSE	60	8	0.1333	8.87E-16	3.36E-13
GO_LEUKOCYTE_MIGRATION	259	11	0.0425	9.58E-16	3.54E-13
PID_IL12_2PATHWAY	63	8	0.127	1.34E-15	4.84E-13
GO_REGULATION_OF_LYMPHOCYTE_MEDIATED_IMMUNITY	114	9	0.0789	1.70E-15	5.99E-13
PID_CD8_TCR_DOWNSTREAM_PATHWAY	65	8	0.1231	1.74E-15	6.02E-13
BIOCARTA_CTL_PATHWAY	15	6	0.4	2.68E-15	9.06E-13
GO_LOCOMOTION	1114	16	0.0144	2.82E-15	9.32E-13
KEGG_LEISHMANIA_INFECTION	72	8	0.1111	4.11E-15	1.24E-12
GSE26495_PD1HIGH_VS_PD1LOW_CD8_TCELL_DN	200	10	0.05	4.24E-15	1.24E-12
GSE3039_NKT_CELL_VS_ALPHAALPHA_CD8_TCELL_DN	200	10	0.05	4.24E-15	1.24E-12

Supplementary Table 4: NLF GSEA

Overlap Results

Collection(s):	C2, C5, C7
# overlaps shown:	50
# genesets in collections:	15527
# genes in comparison (n):	7
# genes in universe (N):	45956

Gene Set Name	# Genes in Gene Set (K)	# Genes in Overlap (k)	k/K	p-value	FDR q-value
SMIRNOV_CIRCULATING_ENDOTHELIOCYTES_IN_CANCER_UP	158	4	0.0253	4.67E-09	4.24E-05
GO_REGULATION_OF_IMMUNE_SYSTEM_PROCESS	1403	6	0.0043	5.46E-09	4.24E-05
PID_SYNDECAN_2_PATHWAY	33	3	0.0909	1.18E-08	4.68E-05
GSE29618_MONOCYTE_VS_MDC_UP	200	4	0.02	1.21E-08	4.68E-05
GO_POSITIVE_REGULATION_OF_RESPONSE_TO_STIMULUS	1929	6	0.0031	3.66E-08	1.07E-04
GO_IMMUNE_SYSTEM_PROCESS	1984	6	0.003	4.33E-08	1.07E-04
GO_POSITIVE_REGULATION_OF_IMMUNE_SYSTEM_PROCESS	867	5	0.0058	4.81E-08	1.07E-04
KEGG_LEISHMANIA_INFECTION	72	3	0.0417	1.28E-07	2.49E-04
GO_CELLULAR_RESPONSE_TO_LIPID	457	4	0.0088	3.30E-07	5.69E-04
KEGG_TOLL_LIKE_RECEPTOR_SIGNALING_PATHWAY	102	3	0.0294	3.69E-07	5.73E-04
GO_ENDOCYTOSIS	509	4	0.0079	5.07E-07	7.16E-04
LENAOUR_DENDRITIC_CELL_MATURATION_DN	128	3	0.0234	7.33E-07	9.48E-04
KEGG_NATURAL_KILLER_CELL_MEDIATED_CYTOTOXICITY	137	3	0.0219	8.99E-07	1.07E-03
GO_CELLULAR_RESPONSE_TO_BIOTIC_STIMULUS	163	3	0.0184	1.52E-06	1.41E-03
MARTINELLI_IMMATURE_NEUTROPHIL_DN	13	2	0.1538	1.55E-06	1.41E-03
GSE6269_HEALTHY_VS_STAPH_PNEUMO_INF_PBMC_DN	169	3	0.0178	1.69E-06	1.41E-03
GO_RESPONSE_TO_EXTERNAL_STIMULUS	1821	5	0.0027	1.91E-06	1.41E-03
GO_RESPONSE_TO_CYTOKINE	714	4	0.0056	1.95E-06	1.41E-03
GO_CELLULAR_RESPONSE_TO_ORGANIC_SUBSTANCE	1848	5	0.0027	2.05E-06	1.41E-03
GO_PHAGOCYTOSIS	190	3	0.0158	2.41E-06	1.41E-03
GO_REGULATION_OF_DEFENSE_RESPONSE	759	4	0.0053	2.48E-06	1.41E-03
XU_RESPONSE_TO_TRETINOIN_AND_NSC682994_UP	17	2	0.1176	2.70E-06	1.41E-03
GSE34156_NOD2_LIGAND_VS_TLR1_TLR2_LIGAND_6H_TREATED_MO	199	3	0.0151	2.76E-06	1.41E-03
GSE12845_PRE_GC_VS_DARKZONE_GC_TONSIL_BCELL_UP	200	3	0.015	2.81E-06	1.41E-03
GSE22886_NAIVE_BCELL_VS_NEUTROPHIL_DN	200	3	0.015	2.81E-06	1.41E-03
GSE22886_NAIVE_CD4_TCELL_VS_MONOCYTE_DN	200	3	0.015	2.81E-06	1.41E-03
GSE22886_NAIVE_CD8_TCELL_VS_MONOCYTE_DN	200	3	0.015	2.81E-06	1.41E-03
GSE22886_NAIVE_TCELL_VS_MONOCYTE_DN	200	3	0.015	2.81E-06	1.41E-03
GSE29618_MONOCYTE_VS_MDC_DAY7_FLU_VACCINE_UP	200	3	0.015	2.81E-06	1.41E-03
GSE29618_MONOCYTE_VS_PDC_DAY7_FLU_VACCINE_UP	200	3	0.015	2.81E-06	1.41E-03
GSE29618_MONOCYTE_VS_PDC_UP	200	3	0.015	2.81E-06	1.41E-03
GO_CELLULAR_RESPONSE_TO_OXYGEN_CONTAINING_COMPOUND	799	4	0.005	3.04E-06	1.48E-03
KEGG_REGULATION_OF_ACTIN_CYTOSKELETON	216	3	0.0139	3.53E-06	1.64E-03
WIERENGA_STAT5A_TARGETS_UP	217	3	0.0138	3.58E-06	1.64E-03
ZHANG_RESPONSE_TO_IKK_INHIBITOR_AND_TNF_UP	223	3	0.0135	3.89E-06	1.73E-03
GO_REGULATION_OF_IMMUNE_RESPONSE	858	4	0.0047	4.04E-06	1.74E-03
GO_RECEPTOR_MEDIATED_ENDOCYTOSIS	231	3	0.013	4.32E-06	1.80E-03
OSWALD_HEMATOPOIETIC_STEM_CELL_IN_COLLAGEN_GEL_UP	233	3	0.0129	4.44E-06	1.80E-03
GO_RESPONSE_TO_BIOTIC_STIMULUS	886	4	0.0045	4.59E-06	1.80E-03
GO_RESPONSE_TO_LIPID	888	4	0.0045	4.63E-06	1.80E-03
GO_REGULATION_OF_POSITIVE_CHEMOTAXIS	24	2	0.0833	5.48E-06	2.08E-03
MCLACHLAN_DENTAL_CARIES_UP	254	3	0.0118	5.75E-06	2.12E-03
PID_LYMPH_ANGIOGENESIS_PATHWAY	25	2	0.08	5.96E-06	2.15E-03
KEGG_CYTOKINE_CYTOKINE_RECEPTOR_INTERACTION	267	3	0.0112	6.67E-06	2.35E-03
PID_TRAIL_PATHWAY	28	2	0.0714	7.50E-06	2.59E-03
GO_POSITIVE_REGULATION_OF_RESPONSE_TO_EXTERNAL_STIMUL	296	3	0.0101	9.08E-06	3.05E-03
GO_LIPOPOLYSACCHARIDE_MEDIATED_SIGNALING_PATHWAY	31	2	0.0645	9.23E-06	3.05E-03
GO_IMMUNE_RESPONSE	1100	4	0.0036	1.08E-05	3.49E-03
GO_RESPONSE_TO_MOLECULE_OF_BACTERIAL_ORIGIN	321	3	0.0093	1.16E-05	3.67E-03
KEGG_PATHWAYS_IN_CANCER	328	3	0.0091	1.23E-05	3.83E-03

Supplementary Table 5: NLF and PB Regression with Age Data

NLF Regression with Age:

Column ID	r	p-value (correlation)	Lower CI	Upper CI	N
GZMB	-0.452535	0.0593381	-0.759043	0.0181847	18
TNF	0.411792	0.0895161	-0.0681971	0.73698	18
CD57	0.399308	0.100668	-0.0830523	0.730079	18
ITGA2	0.364479	0.137001	-0.123396	0.71046	18
MIP-1a	0.350301	0.154119	-0.139371	0.702316	18
KIR2DS2	0.334912	0.174297	-0.156424	0.693372	18
KLRD1	0.32891	0.182628	-0.162997	0.689853	18
ITGAL	-0.31705	0.199863	-0.68285	0.175859	18
IL18R1	-0.316456	0.200753	-0.682497	0.176498	18
ITGAM	-0.316394	0.200847	-0.68246	0.176565	18
IL8	0.315172	0.202687	-0.17788	0.681734	18
IFNGR1	-0.292431	0.238967	-0.668094	0.202029	18
CD244	-0.287809	0.246815	-0.66529	0.206865	18
PTPN11	0.285618	0.250591	-0.209149	0.663958	18
CD69	-0.277421	0.26504	-0.65895	0.217646	18
TYROBP	-0.274058	0.271117	-0.656886	0.221111	18
NCAM1	0.250559	0.315943	-0.244976	0.6423	18
TNFSF10	-0.247695	0.321689	-0.640503	0.247844	18
NCR2	0.247203	0.322682	-0.248336	0.640193	18
CD14	-0.24579	0.325546	-0.639304	0.249747	18
CXCR3	0.232373	0.353466	-0.263042	0.630814	18
FCGR2B	-0.213443	0.395095	-0.618669	0.281485	18
ITGB2	-0.212863	0.396411	-0.618294	0.282044	18
KIR3DL2	0.208685	0.405961	-0.286063	0.615586	18
KIR2DL4	-0.198135	0.430621	-0.608704	0.296136	18
HCST	0.197434	0.432287	-0.296802	0.608245	18
MAPK1	-0.188222	0.454487	-0.602182	0.305501	18
NCR1	0.184308	0.464094	-0.309174	0.599591	18
SH2D1B	0.181409	0.471272	-0.311883	0.597667	18
RANTES	0.181218	0.471748	-0.312062	0.597539	18
IL22	-0.177456	0.481149	-0.595035	0.315566	18
MAPK3	-0.16287	0.518457	-0.585245	0.329027	18
CXCL10	0.150906	0.55003	-0.33992	0.577122	18

GNLY	0.139945	0.579683	-0.349787	0.569606	18
IFNAR2	-0.137384	0.586708	-0.567839	0.352076	18
IL13	-0.133741	0.596758	-0.56532	0.355323	18
IFNAR1	0.122283	0.628829	-0.365459	0.557341	18
IFNGR2	-0.11834	0.640015	-0.554577	0.36892	18
JAK1	-0.117456	0.642532	-0.553956	0.369694	18
CD247	-0.110862	0.661434	-0.549307	0.375448	18
PTPN6	-0.108749	0.667532	-0.547813	0.377284	18
IL12RB1	0.108258	0.668953	-0.37771	0.547465	18
KLRC2	0.103644	0.682349	-0.381704	0.544188	18
KLRC1	0.102246	0.686426	-0.38291	0.543193	18
NFATC2	0.102055	0.686983	-0.383075	0.543057	18
JAK2	0.0996597	0.693989	-0.385138	0.541349	18
STAT1	0.0822892	0.745476	-0.399954	0.528849	18
ITGAX	0.0820019	0.746337	-0.400197	0.52864	18
CSF2	0.0803021	0.751436	-0.401633	0.527406	18
IL4	0.0794931	0.753867	-0.402316	0.526818	18
FCGR3A	-0.0761203	0.764023	-0.524362	0.405156	18
STAT5B	-0.0652754	0.796922	-0.516415	0.414226	18
IL17A	0.0646929	0.798699	-0.41471	0.515986	18
KLRC3	0.0628675	0.804274	-0.416227	0.514639	18
PRF1	-0.0597877	0.813698	-0.512363	0.418779	18
FCGR2A	0.05879	0.816757	-0.419605	0.511624	18
KLRB1	-0.0578508	0.819639	-0.510928	0.420381	18
KLRK1	-0.0528939	0.834882	-0.507245	0.424466	18
FCER1G	0.0506559	0.841783	-0.426304	0.505576	18
NCR3	-0.0479707	0.850078	-0.50357	0.428504	18
STAT5A	0.0367475	0.884899	-0.437638	0.49513	18
FASLG	-0.0347734	0.891047	-0.493637	0.439235	18
MIP-1b	-0.0327947	0.897214	-0.492137	0.440832	18
IFNG	0.0319352	0.899895	-0.441525	0.491485	18
IL5	-0.0297776	0.906629	-0.489845	0.443262	18
NR0B2	-0.0222161	0.930275	-0.484073	0.449322	18
KIR2DL2	-0.0193114	0.939375	-0.481844	0.451638	18

PB Regression with Age:

Column ID	r	p-value(correlation)	Lower CI	Upper CI	N
IFNG	0.5209	0.0266534	0.0713821	0.794546	18
CD57	0.411423	0.0898322	-0.0686391	0.736777	18
MAPK1	-0.370032	0.130673	-0.713624	0.117071	18
TNF	0.349231	0.155467	-0.140565	0.701698	18
TYROBP	-0.340705	0.166503	-0.696752	0.150038	18
IFNGR1	-0.339286	0.16839	-0.695925	0.151607	18
NCR1	-0.329512	0.181781	-0.690207	0.16234	18
NR0B2	-0.322611	0.191653	-0.686142	0.169849	18
FASLG	0.321691	0.192996	-0.170846	0.685598	18
KIR2DS2	-0.314769	0.203297	-0.681495	0.178313	18
IL8	-0.30459	0.219088	-0.675419	0.189191	18
NCAM1	-0.297781	0.230084	-0.671326	0.196401	18
MAPK3	-0.295532	0.233792	-0.669969	0.198772	18
STAT5B	-0.294479	0.235541	-0.669333	0.199879	18
KLRK1	-0.289073	0.244652	-0.666058	0.205545	18
GNLY	0.27591	0.26776	-0.219205	0.658023	18
NCR2	-0.267652	0.282924	-0.652938	0.227676	18
KIR3DL2	-0.265072	0.287768	-0.651342	0.230308	18
FCGR2B	0.262126	0.293358	-0.233303	0.649515	18
STAT5A	-0.258291	0.300734	-0.647131	0.237189	18
IL18R1	-0.253253	0.310593	-0.643987	0.24227	18
IL4	-0.251032	0.314998	-0.642597	0.244501	18
IFNAR2	-0.24326	0.330708	-0.637711	0.252268	18
JAK1	-0.241596	0.33413	-0.636661	0.253923	18
CD14	-0.237944	0.341711	-0.634351	0.257544	18
IL12RB1	-0.231456	0.355423	-0.63023	0.263944	18
RANTES	0.229127	0.360421	-0.266231	0.628745	18
FCGR3A	0.216332	0.388575	-0.278693	0.620535	18
NCR3	-0.208831	0.405626	-0.61568	0.285924	18
ITGA2	-0.201221	0.423328	-0.610724	0.293201	18
ITGAX	-0.197207	0.432829	-0.608096	0.297018	18
MIP-1b	0.192985	0.442937	-0.301013	0.605323	18
ITGAL	0.18682	0.457916	-0.306818	0.601255	18
FCGR2A	-0.171668	0.495795	-0.591165	0.320931	18
KLRC2	-0.168262	0.504512	-0.588878	0.324074	18
CD69	-0.164755	0.51356	-0.586517	0.327298	18

KLRC1	-0.159715	0.526702	-0.583111	0.331913	18
SH2D1B	-0.148147	0.557429	-0.575237	0.342414	18
STAT1	-0.139564	0.580726	-0.569343	0.350128	18
KIR2DL2	-0.132291	0.600779	-0.564314	0.356612	18
TNFSF10	-0.119847	0.635729	-0.555635	0.367599	18
CSF2	0.11912	0.637795	-0.368236	0.555125	18
CXCR3	0.111495	0.659609	-0.374897	0.549755	18
IFNGR2	-0.108728	0.667591	-0.547798	0.377302	18
HCST	-0.0983635	0.69779	-0.540423	0.386252	18
CD247	-0.0918039	0.717131	-0.53572	0.391869	18
IL5	-0.0901598	0.722005	-0.534537	0.393272	18
PRF1	0.0762484	0.763637	-0.405048	0.524456	18
KLRC3	0.0738671	0.770829	-0.407048	0.522718	18
IL22	0.0737459	0.771195	-0.40715	0.522629	18
KIR2DL4	0.0708074	0.780096	-0.409611	0.520479	18
ITGB2	-0.0550689	0.828186	-0.508863	0.422676	18
PTPN11	-0.0519474	0.837799	-0.50654	0.425243	18
JAK2	0.0513407	0.83967	-0.425742	0.506087	18
ITGAM	-0.0511354	0.840304	-0.505934	0.42591	18
IFNAR1	0.050728	0.841561	-0.426244	0.50563	18
NFATC2	-0.0479284	0.850209	-0.503538	0.428538	18
KLRB1	-0.0434713	0.864009	-0.500197	0.432177	18
KLRD1	0.0403738	0.873623	-0.434697	0.497867	18
CD244	-0.0380531	0.880837	-0.496116	0.436581	18
IL13	-0.0375488	0.882405	-0.495736	0.436989	18
GZMB	0.0346303	0.891492	-0.439351	0.493528	18
PTPN6	0.0184039	0.942219	-0.45236	0.481147	18
FCER1G	-0.0118122	0.962897	-0.476064	0.457589	18
MIP-1a	0.0114587	0.964007	-0.457868	0.475791	18
IL17A	0.00223803	0.992968	-0.465125	0.468626	18
CXCL10	-0.0015145	0.995241	-0.468061	0.465692	18
